# A stochastic quantum program synthesis framework based on Bayesian optimization

**DOI:** 10.1038/s41598-021-91035-3

**Published:** 2021-06-23

**Authors:** Yao Xiao, Shahin Nazarian, Paul Bogdan

**Affiliations:** 1 University of Southern California, Los Angeles, CA 90089 USA; 2 Department of Electrical and Computer Engineering, Viterbi School of Engineering, University of Southern California, Los Angeles, CA 90089 USA

**Keywords:** Computer science, Quantum physics

## Abstract

Quantum computers and algorithms can offer exponential performance improvement over some NP-complete programs which cannot be run efficiently through a Von Neumann computing approach. In this paper, we present BayeSyn, which utilizes an enhanced stochastic program synthesis and Bayesian optimization to automatically generate quantum programs from high-level languages subject to certain constraints. We find that stochastic synthesis can comparatively and efficiently generate a program with a lower cost from the high dimensional program space. We also realize that hyperparameters used in stochastic synthesis play a significant role in determining the optimal program. Therefore, BayeSyn utilizes Bayesian optimization to fine-tune such parameters to generate a suitable quantum program.

## Introduction

We have reached an era where the fundamental physical size limits of CMOS based transistors have dampened the future of computing. Researchers have investigated the new non-silicon non-Von Neumann architectures^1^ such as neuromorphic and quantum computing^2,3^*.* Especially in quantum computing, quantum supremacy has been recognized as the goal of demonstrating that a quantum device can solve a problem which classical ones cannot solve efficiently^4^. Quantum computers and algorithms^5^ such as Shor’s algorithm^6^ can offer exponential performance improvement over some NP-complete programs which cannot be run efficiently through a Von Neumann computing approach. However, there are some impediments to scientific advances in quantum computing and algorithms^7,8^. First, while there are some quantum programming languages^9–12^*,* it is still a burden for programmers without a basic understanding of quantum computing to write quantum programs. Second, instead of mapping computational tasks onto general-purpose quantum processors, one needs to determine how to automatically synthesize quantum accelerators^13,14^ given an application.

**Figure 1:**
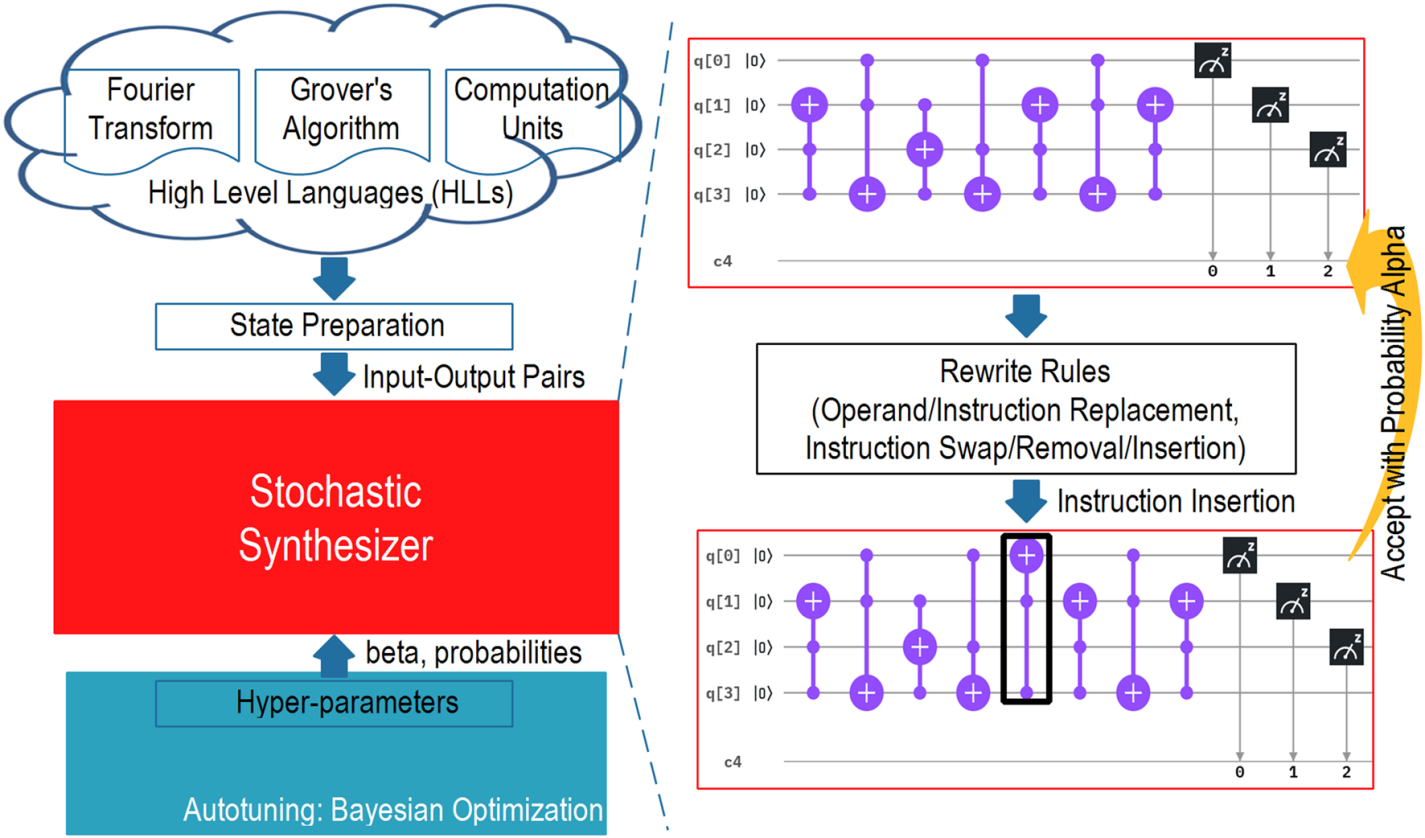
The overview of the automatic quantum program synthesis from high-level languages. We first prepare input–output pairs from high-level languages. These pairs guide the stochastic synthesizer with hyper-parameters to search towards the quantum program with the lowest cost to best fit them. Inside the synthesizer, a quantum program is initially proposed at random. Followed by acting on randomly chosen rules such as replacement, insertion, and deletion, a new proposed program is generated and accepted with a probability inversely proportional to the cost. This process is repeated until either the synthesizer finds the correct program, or the budget is exhausted.

Therefore, rather than compiling quantum circuits^15^ from quantum programs, in this paper, we aim to provide a mathematical and algorithmic framework that is capable of automatically designing quantum circuits/accelerators from high-level languages such as C/C++ that are familiar to programmers. Specifically, we have developed a stochastic synthesis^16,17^ in program super-optimization for × 86–64 to quantum programs. Instead of focusing on quantum compilation optimization, we modify the techniques to synthesize quantum circuits from high-level languages by applying input–output pairs obtained from them (c.f. Fig. 1). The experimental results provide three important observations: Firstly, the total error between golden results and synthesized results sometimes remains the same even if one operand is replaced with another. Hence, considering only the synthesis error in the cost function can lead to a constant acceptance of a proposed program without providing sufficient exploration of similar programs in a high dimensional space. Secondly, the hyper-parameters used in stochastic synthesis dominate the performance efficiency and outcome of the program. In practice, it is extremely difficult for humans to fine-tune such parameters. However, the proposed BayeSyn aims for optimality^18,19^ by fine-tuning hyperparameters and achieves highly efficient results in terms of area and power consumption. Thirdly, final local refinements are more challenging to achieve than initial global refinements, i.e. first several thousand iterations can reach a low cost rapidly, however, further refining of the cost towards the optimality is extremely challenging as it would take tens or hundreds of thousand iterations. Next, we will present how BayeSyn effectively deals with such scenarios.

## Stochastic quantum program synthesis from HLLs

To minimize the burdens on the programmers and improve programming efficiency, we develop a stochastic synthesis to generate a series of quantum gates that perform exact tasks guided by high-level languages (HLLs) as shown in Fig. 1. Our synthesizer generates a random or exhaustive set of testcases (input–output pairs) by analyzing HLLs written in C or C++ , and a set of hyperparameters used for the synthesis. It first randomly synthesizes a program of a given length. Next, it iteratively generates new programs by randomly selecting the rewrite rules, and it improves the search by generating a program that performs better under a set of testcases. The goal of the BayeSyn is to synthesize a corresponding quantum program by drawing an optimal element from a probability density function (PDF) based on an input application. It guarantees that regions of higher probability are sampled more often, which allows the synthesizer to locally refine and search for the optimized program.

Each program is described in an irregular and high dimensional space and is associated with a cost function to capture different requirements such as correctness, soundness, and resource efficiency. In this paper, we focus on the correctness of quantum programs and performance efficiency of the synthesizer by designing a cost function as follows:

$$C\left( P \right) = {\mathop \sum \limits_{{i = 1}}^{n} \left[ {a\left( {P{\text{|}}t_{i} } \right) - g\left( {t_{i} } \right)} \right]} + {\mathop \sum \limits_{{i = 1}}^{n} 1\left( {a\left( {P{\text{|}}t_{i} } \right) \ne g\left( {t_{i} } \right)} \right)}$$

where *C*(*P*) is the total cost associated with a program *P*; $a\left(P|{t}_{i}\right)$ is an actual result of the program *P* under the *i-*th testcase; $g\left({t}_{i}\right)$ is a golden result from input–output pairs; $1\left(s\right)$ is the indicator function, which equals 1 if *s* is true, and 0 otherwise. Therefore, the first term measures the total sum of differences between actual results and golden results, which helps BayeSyn to search for a cost-optimized program. However, as learned from experiments, sometimes a synthesizer may choose a rewrite rule which acts on some correlated operands to generate a new program ${P}^{*}$ from *P*. This rule makes the first term identical for *P* and ${P}^{*}$, causing the synthesizer to always accept the new proposal ${P}^{*}$ without fully exploring local refinements of *P*. The random walk phenomenon is carefully prevented in BayeSyn by the second term which measures the number of failed testcases. This helps BayeSyn search for a better program ${P}^{*}$, which is in the proximity of the old program *P* in a high dimensional space. One approach is to convert any cost function into a PDF as follows:

1 $$p\left(P\right)=\frac{1}{Z}{e}^{-\beta \bullet C\left(P\right)}$$

where *Z* is a normalizing term and *β* is a hyperparameter to tune.

A new proposed program ${P}^{*}$ with rewrites from the synthesizer is either rejected or accepted with a probability α. If it is accepted, ${P}^{*}$ becomes the current program used for the next iteration. Otherwise, BayeSyn continues to explore the optimized program from the old program *P*. This acceptance probability^20,21^ is designed as follows

2 $$\alpha \left(P\to {P}^{*}\right)=min\left(1,\frac{p\left({P}^{*}\right)q\left(P|{P}^{*}\right)}{p\left(P\right)q\left({P}^{*}|P\right)}\right)$$

where $p\left(\bullet \right)$ comes from Eq. (1); $q({P}^{*}|P$) is the proposed distribution from which ${P}^{*}$ is sampled based on *P*.

In our stochastic program synthesis, we design probabilities of transforming one program into another in such a way that probabilities of rewrite rules are the same as those of undoing rules. Therefore, Eq. (2) can be simplified as

3 $$\alpha \left(P\to {P}^{*}\right)=min\left(1,\frac{p\left({P}^{*}\right)}{p\left(P\right)}\right)$$

$$=min\{1,{e}^{-\beta [C({P}^{*})-C(P)]}\}$$

The rewritten program ${P}^{*}$ is always accepted (i.e., $\alpha \left(P\to {P}^{*}\right)=1$) if it is better (i.e., $C({P}^{*})<C(P)$) compared to *P*. With a small probability $\alpha$, it can still be accepted if it is worse, to prevent the search from falling into local optima. Occasional acceptance of worse proposals can help the search jump out of local optima. In addition, $\beta$ plays a significant role in choosing the optimal solution. If $\beta$ is too small, the search follows a random walk where each proposed program is accepted. However, if $\beta$ is too large, the search becomes local hill climbing where finding the global optimum cannot be guaranteed. Therefore, we discuss next, an approach to autotune such parameters.

A new program ${P}^{*}$ is proposed from *P* based on rewrite rules. We design a set of rewrite rules in such a way to globally adjust program structures such as instruction swap and deletion, and locally refine partially correct programs such as operand replacement. Each rule is assigned with a probability to determine how often it is selected in the synthesizer. However, compared to all existing program synthesizers in the literature, we adaptively adjust these probabilities to make sure that in the beginning, frequent global modifications can quickly find an optimum; in the end, frequent local refinements can perturb programs to reach the optimum. In the implementation, we choose the following rules to act on programs:

1. Replace an operand: Randomly select an instruction from the quantum program, and randomly pick one of its operand (qubits). With probability ${p}_{ro}$, the operand is replaced with a new randomly generated operand from a set of available qubits.
2. Replace all operands: Randomly select an instruction from the quantum program. With probability ${p}_{rao}$, all the operands are replaced with new randomly generated operands from a set of available qubits.
3. Replace a gate: Randomly select an instruction from the quantum program. With probability ${p}_{rg}$, the gate is replaced with a new randomly generated gate from a set of available gates in a universal quantum gate set. However, this must satisfy that the number of required qubits from the old gate is the same as that of the new one. There are different types of universal sets. In our implementation, we use the Toffoli and Hadamard gates as a universal quantum gate set.
4. Replace an instruction: Randomly select an instruction from the quantum program. With probability ${p}_{ri}$, the instruction (gate plus operands) is replaced with a new randomly generated instruction.
5. Swap two instructions: Two instructions are randomly selected and with the probability ${p}_{si}$, the two instructions are swapped.
6. Insert an instruction: Randomly select an instruction $i$ from the quantum program. With probability ${p}_{ii}$, a new instruction is randomly generated and inserted after the instruction $i$.
7. Delete an instruction: Randomly select an instruction from the quantum program. With probability ${p}_{di}$, this instruction is removed from the program.

All of the probabilities (i.e., ${p}_{ro}$, ${p}_{rao}$, ${p}_{rg}$, ${p}_{ri}$, ${p}_{si}$, ${p}_{ii}$, and ${p}_{di}$) are considered as hyperparameters. While traditionally in the literature, these hyperparameters are tuned by humans through a very time-consuming process, we introduce a Bayesian optimization approach to autotune them. In contrast to prior work, we adaptively vary these probabilities during program synthesis. For example, a low cost means that a generated quantum program is globally almost correct, but requires local refinements. Therefore, we increase the probabilities of ${p}_{ro}$ and ${p}_{rg}$ and lower the rest of the probabilities.

**Figure 2:**
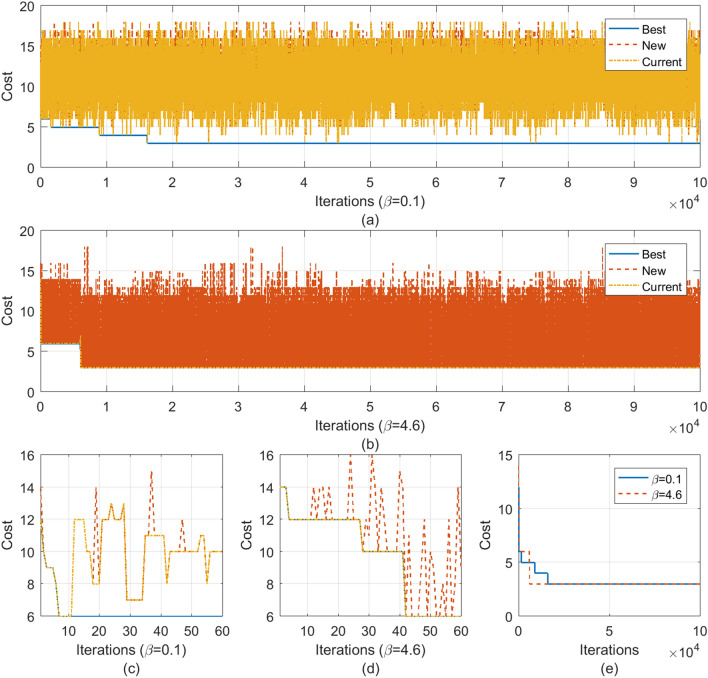
Experimental results on stochastic quantum program synthesis. (**a**) shows the average cost of the best, new, and current programs when *β* equals 0.1. Small *β* means random exploration, which can be demonstrated by the current program cost. (**b**) shows the average cost of the best, new, and current programs when *β* equals 4.6. Large *β* means hill climbing, which can be demonstrated by the current program cost, which is equal to the best program cost. (**c**,**d**) show the first 60 iterations to illustrate the variation of different costs. (**e**) compares the best program costs when *β* equals 0.1 and 4.6, which validates that *β* plays a partial role in the convergence of stochastic synthesis*.*

We applied different benchmarks to demonstrate the validity of our BayeSyn framework: the quantum adder, multiplier, Grover’s algorithm, and Shor’s algorithm. As shown in Fig. 2, we measure the average costs of the current, new, and best programs for different $\beta$ values. Figure 2a shows different costs when $\beta =0.1$. The current program cost is randomly distributed compared to the best program cost, which validates that small $\beta$ allows the synthesizer to randomly explore the program space. Figure 2b, instead, shows different costs when $\beta =4.6$. The current program cost is the same as the best program cost. This is because large $\beta$ means hill climbing, which guides the synthesizer to always follow the best move. Figure 2c,d demonstrate the trend of different program costs for the first 60 iterations. Figure 2e compares the best program costs when $\beta =0.1$ and 4.6. This validates our statement that $\beta$ plays a partial role for the synthesizer in quickly converging to the optimal program.

## Autotuning hyperparameters for efficient quantum program synthesis

As discussed previously, the hyperparameters such as $\beta$ and the probabilities (i.e., ${p}_{ro}$, ${p}_{rao}$, ${p}_{rg}$, ${p}_{ri}$, ${p}_{si}$, ${p}_{ii}$, and ${p}_{di}$) determine the final optimal quantum program. Of note, $\beta$ is different for different applications. It is extremely difficult for humans to tune such parameters. Therefore, we propose a Bayesian optimization (BO) to autotune parameters. The goal of this BO approach is to find the extrema of black-box objective functions that are expensive to evaluate, but cheap to sample^18,19^*.* BO requires two components: (1) prior beliefs and likelihood to guide the sampling and derive the posterior; (2) the acquisition function to trade off exploration and exploitation of the search space. Priors capture our beliefs on properties of the black-box objective function such as smoothness and extrema locations. The acquisition function determines where to sample next to minimize the number of evaluations.

The black-box objective function is the accuracy of the current program on a set of testcases. The domain *X* is a high dimensional space of ($\beta$, probabilities) values. The goal is to find the hyperparameters to maximize the accuracy. We use the Beta distribution rather than the Gaussian distribution as prior knowledge concerning the probability of success in sampling:

4 $$f\left(x;\alpha ,\beta \right)=\frac{1}{B\left(\alpha ,\beta \right)}{x}^{\alpha -1}{\left(1-x\right)}^{\beta -1}$$

where $B\left(\alpha ,\beta \right)=\frac{\Gamma (\alpha +\beta )}{\Gamma (\alpha )\Gamma (\beta )}$ is a normalization factor and $\Gamma \left(\alpha \right)$ is the Gamma function. When $\alpha =\beta =2$, it reduces to Gaussian. However, the improvement of the Beta distribution is that it can control where to sample more often by adjusting $\alpha$ and $\beta$. Especially the distribution is skewed when $\alpha$ isn’t equal to $\beta$. The expected improvement is the acquisition function:

5 $${\varphi }_{t}\left(x\right)=E\left(max\left\{0,{f}_{t+1}\left(x\right)-f\left({x}^{+}\right)\right\}|{D}_{t}\right)$$

where *f* represents the black-box function and ${x}^{+}={argmax}_{{x}_{i}\in {x}_{1:t}}f({x}_{i})$.

**Figure 3:**
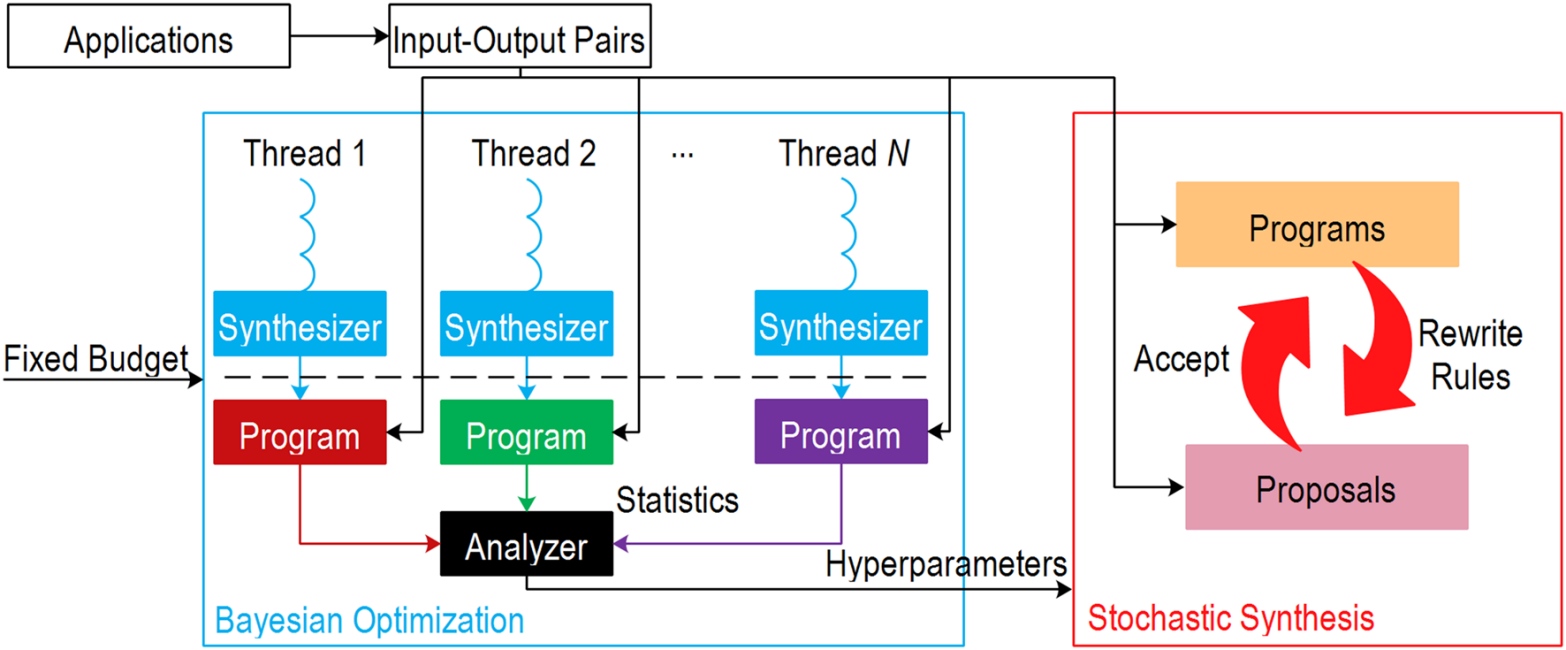
Bayesian Optimization (BO) to fine-tune hyper-parameters. Instead of randomly guessing the hyper-parameters, a BO approach systematically searches for the best parameters. First, we run several synthesizers in parallel with a fixed budget (e.g., 10 min or 10,000 iterations) and input–output pairs to collect statistics (the cost and the number of failed testcases), which are further used to guess the better parameters for the next iteration by the acquisition function. Finally, the best parameters are applied to the final stochastic synthesizer to generate the quantum program.

Figure 3 illustrates the implementation of BO on stochastic synthesis. We give a fixed budget to each synthesizer such as one hour with different hyperparameters. After the budget is exhausted, we collect statistics such as the number of failed testcases and the total error, combined with hyperparameters into ${D}_{t}$ used in Eq. (5). Next, we use the acquisition function to decide how to choose the next set of hyperparameters to guide the search for the optimum of the objective function.

**Figure 4:**
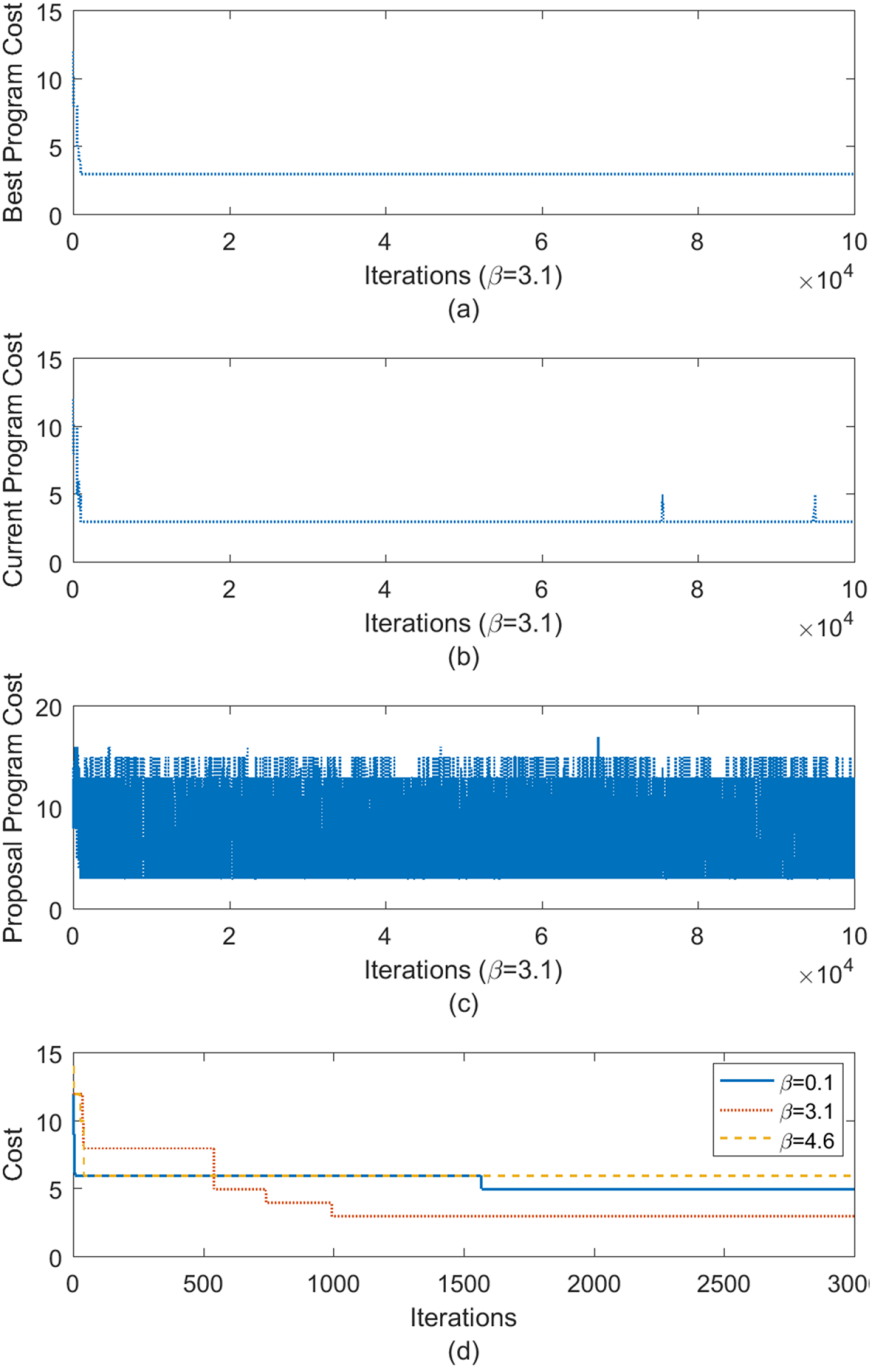
Parameter fine-tuning using Bayesian optimization. We use Bayesian optimization to auto-tune parameters such as *β* and probabilities as they determine the performance of a synthesizer. We find out that $\beta =3.1$ is a better choice compared to $\beta =0.1$ and 4.6. (**a**–**c**) show the best, current, and proposal program costs, respectively. It is interesting to see that a few bumps exist in the current program cost. This is because the stochastic nature allows the synthesizer to accept a possibly worse case to explore. (**d**) compares the best program costs of different *β* values. $\beta =3.1$ can quickly find a better program at a low cost compared to others.

Experimental results shown in Fig. 4 illustrate the results obtained with the above-mentioned BO approach to auto-tune the parameters (e.g., *β*) as they determine the performance of a synthesizer. We find that $\beta =3.1$ is a better choice compared to $\beta =0.1$ or 4.6. Figures 4a–c show the best, current, and proposed program costs, respectively. It is interesting to see that the trend of the current program cost in Fig. 4b sits between Fig. 2a,b. In general, it follows the pattern of hill climbing as it continues to explore the program space to reject programs with large costs. However, occasionally, the synthesizer accepts a worse program (demonstrated by a few bumps in Fig. 4b) to explore a different region. That is, the synthesizer with $\beta =3.1$ combines the random search (small $\beta$) and hill climbing (large $\beta$). In addition, Fig. 4d compares the best program costs for different $\beta$ values. $\beta =3.1$ can quickly find a better program at a low cost compared to others.

## Feedback directed search to accelerate quantum program synthesis

Local refinements require too many iterations for the cost function to reach to zero because of infinite possible rewrite rules to act on a program. For example, from experiments, we notice that it only takes 1000 iterations from the total cost to reduce from 20 down to 7, but the synthesizer spends about 10^7^ ~ 10^8^ iterations to make the program correct (zero cost). This issue makes stochastic synthesis far from practical in quantum programming.

**Figure 5:**
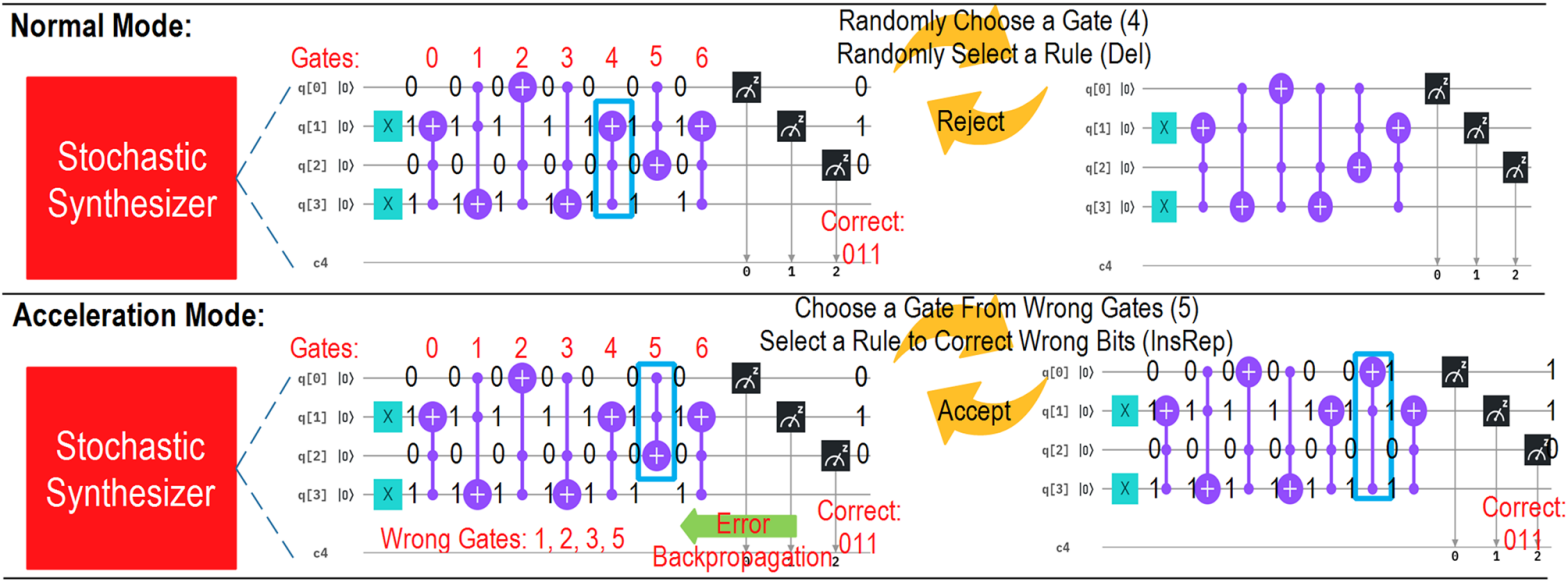
Two modes in stochastic synthesis. In the normal mode, a gate is randomly replaced with another gate. Without knowing the erroneous gates, the new proposed program usually leads to rejection. This may end up using thousands of iterations exploring the wrong regions. However, in the acceleration mode where it is important to locally refine a program without globally dramatically modifying the structure of the program, we calculate the error and backpropagate it to find the erroneous gates. We replace a gate in this set with another to increase the probability of searching for a better program. For example, in this Fig., instead of choosing an irrelevant gate 4, we replace gate 5 and the proposed program is accepted.

To reduce the number of iterations required during local explorations, we propose a feedback-directed search within the stochastic synthesis. The general idea is that whenever a mismatch in the outcome occurs, the error is backpropagated to find a set of wrong wired quantum gates. This information helps the synthesizer to randomly select and adjust a gate from this set, reducing many iterations where unrelated gates are selected and evaluated. For example, Fig. 5 compares both the normal mode and the acceleration mode. In the normal mode, the synthesizer may delete the fourth quantum gate. However, since this rewrite rule does not improve the cost, in the end, the proposed quantum program is rejected. In the acceleration mode, an error is backpropagated and the synthesizer selects the fifth gate from the set of wrong gates. Eventually, the wrong gate is corrected, and the proposed program is accepted.

**Figure 6:**
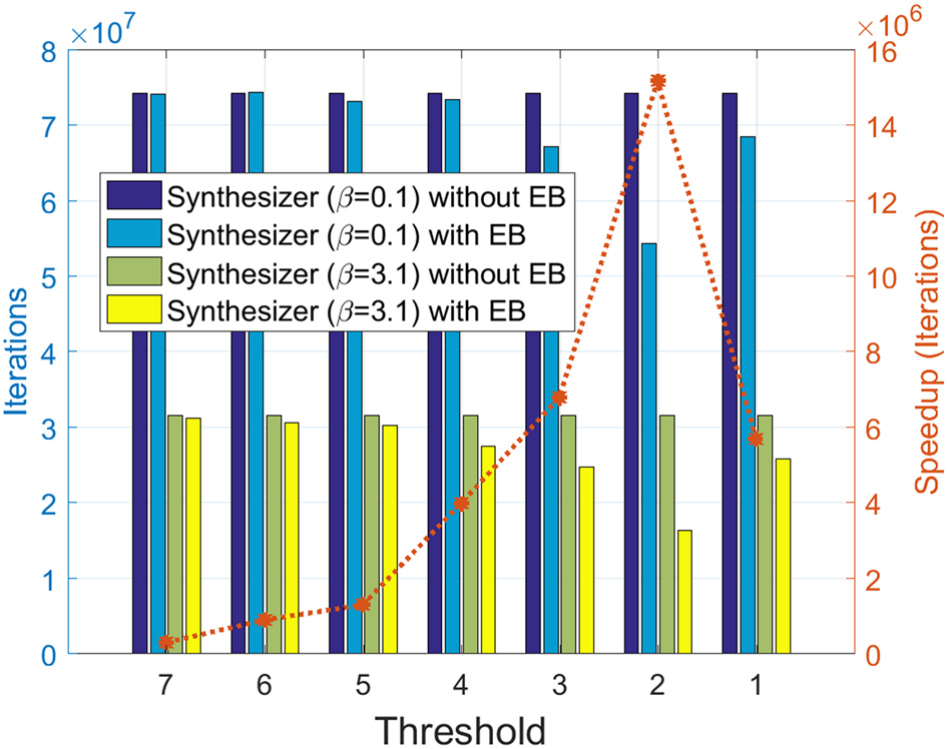
Performance efficiency of error backpropagation (EB). EB allows the synthesizer to replace a known faulty gate rather than guessing it at random. We show the number of iterations and the corresponding speedup (in terms of iterations) for different synthesizers while varying the threshold. The threshold is used to differentiate the normal mode and acceleration mode. If the threshold is too large, the speedup is unnoticeable as the wrong gate list contains all the gates. Replacing a gate from this list is not different from randomly selecting it. However, if the threshold is too small, it is not effective as the synthesizer takes a long time to reach the acceleration mode from the normal mode.

Figure 6 shows the performance efficiency of error backpropagation (EB). EB allows the synthesizer to replace a known faulty gate rather than guessing it at random. We show the number of iterations and the corresponding speedup (in terms of iterations) for different synthesizers while varying the threshold. The threshold is used to differentiate the normal mode and acceleration mode. If the threshold is too large, the speedup is unnoticeable as the wrong gate list contains all the gates. Replacing a gate from this list is not different from randomly selecting it. However, if the threshold is too small, it is not effective as the synthesizer takes a long time to reach the acceleration mode from the normal mode.

## Discussion

**Figure 7:**
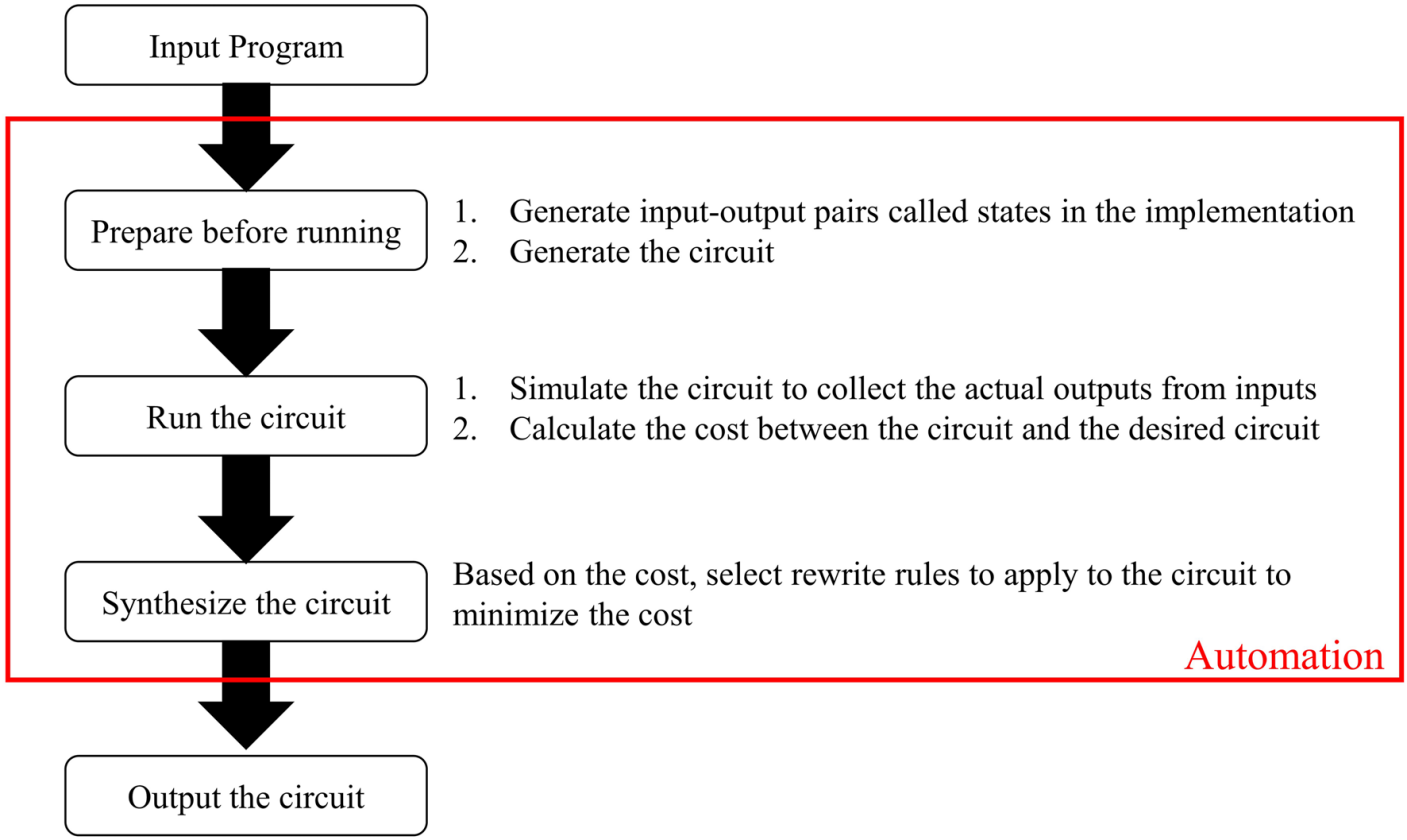
High-level workflow of the framework. The input to the framework is a program written in conventional high-level languages such as C or C++ . Next, the framework prepares input–output pairs to represent this program and randomly generate a circuit. Then, the circuit is simulated to collect actual outputs from inputs and calculate the cost. Finally, based on the cost, rewrite rules are selected, and the acceptance probability is calculated to find the correct circuit implementation as the output of the framework.

We have demonstrated that the stochastic synthesis of our BayeSyn framework is a promising technique to automatically synthesize quantum logic gates from high-level languages. Figure 7 shows a high-level workflow of the framework. The requirement of the framework is to have a working C/C +  + code which can be compiled and executed in standard computers. Next, the framework explores the design space and selects the one that meets our needs. Finally, the framework outputs the circuit. Therefore, compared with Qiskit and some other quantum programming languages, one possible input program to our framework is a simple C code. Note that programmers in this case do not need to know quantum computing to generate a circuit. However, the input program to Qiskit could be related to quantum operations. Therefore, we believe the proposed BayeSyn provides a fundamental path towards full automation in quantum computing.

However, there are several future research directions that require further consideration in order to improve the quality of this quantum program synthesis.

This tool calls for a formal symbolic validator to speed up the run-time execution. Imagine a case where a system requires 32 qubits, which means that ${2}^{32}$ testcases are required in order to synthesize a quantum circuit to fully function under different corner cases. However, if a formal validator such as KLEE^22^ used in × 86–64 assembly is proposed, the synthesis is only checked once, regardless of the number of qubits used in a system. Therefore, the validator provides a formal and efficient approach to reason about the target circuits.

Here, we assume that we have some rewrite rules known to us, but we don't know if these rules work well or there is another set of rewrite rules, which work better. In theory, we can assume that rewrite rules may not be available in advance. The idea is to develop new machine learning techniques (learning to optimize) to find the best rewrite rules that work for quantum computing to maximize the overall performance without sacrificing the correctness of the circuits.

Instead of using stochastic synthesis, some machine learning algorithms offer promising results in program synthesis such as (recurrent) neural networks, reinforcement learning, and generative adversarial networks (GANs). For example, recently GANs are used to synthesize images or tasks from what have previously learned to offer improvements over previous techniques. We believe that GANs can also be used as a promising approach to synthesize quantum programs.

## Data Availability

The prototype of BayeSyn is available from https://github.com/xiaoyao0512/BayeSyn.
